# Phospholipid Nanoparticles: A Novel Colloid for Blood Volume Replacement, Reanimation, and Organ Protection in Hemorrhagic Shock

**DOI:** 10.3390/biomedicines12122824

**Published:** 2024-12-12

**Authors:** Philemon Shallie, Nathan Carpenter, Prashanth Anamthathmakula, Danielle Kinsey, Michael Moncure, Houman Honaryar, Hanieh Sadat Ghazali, Zahra Niroobakhsh, Juan Rodriguez, Cuthbert O. Simpkins

**Affiliations:** 1 Department of Surgery, School of Medicine, University of Missouri Kansas City, Kansas City, MO 64108, USA; 2 Department of Surgery, University Health Truman Medical Center, Kansas City, MO 64108, USA; 3 School of Computing and Engineering, University of Missouri, Kansas City, MO 64112, USAniroobakhshz@umkc.edu (Z.N.); 4 Department of Basic Sciences, University of Health Sciences and Pharmacy, St. Louis, MO 63110, USA

**Keywords:** phospholipid nanoparticles, hemorrhagic shock, resuscitation, nitric oxide, regulation, ischemia–reperfusion injury, oxidative DNA damage, tissue perfusion

## Abstract

**Background/Objectives**: Exsanguination is a leading cause of preventable death in military and civilian settings due to extensive blood loss and hemorrhagic shock, which trigger systemic effects such as impaired tissue perfusion, hypoxia, inflammation, and multi-organ dysfunction. Standard resuscitation restores blood volume but fails to address critical aspects of hemorrhagic shock, including inflammation, coagulopathy, and reperfusion injury. To address these limitations, novel phospholipid nanoparticle (PNP)-based resuscitative fluids, VBI-S and VBI-1, were developed to modulate nitric oxide (NO) levels, improving hemodynamic stability, tissue oxygenation, and reducing inflammatory injury. This study assessed the potential of novel phospholipid nanoparticle fluids, VBI-S and VBI-1, as resuscitative agents for severe hemorrhagic shock by evaluating their ability to regulate nitric oxide, restore blood pressure, and mitigate ischemia–reperfusion injury. **Methods:** This study involved two phases with Sprague Dawley rats (n = 6 per group). Phase one, lasting 4 h, included four groups: blood, Ringer’s lactate, VBI-S, and VBI-1. Phase two, lasting 12 h, comprised sham, blood, and VBI-1 groups. Under anesthesia, one femoral artery was catheterized for blood pressure monitoring, and blood withdrawal from the other induced apnea. Reanimation was performed using an intra-arterial infusion of shed blood, Ringer’s lactate, VBI-S, or VBI-1. Tissue samples were analyzed histologically and for oxidative DNA damage via immunofluorescence. Chemiluminescence and rheology assessed nitric oxide interactions and viscosity. Data were analyzed using ANOVA. **Results**: VBI-1 and shed blood increased mean arterial pressure (MAP) from <10 mmHg to survivable levels sustained for 12 h, with VBI-1 showing significantly higher MAP at 3–4 h. Rats treated with Ringer’s lactate died within 30 min. Histology revealed reduced organ damage in VBI-1-treated rats compared to shed blood. Immunohistochemistry indicated significantly less oxidative DNA damage (*p* < 0.001) in VBI-1-treated rats. VBI-1 exhibited superior viscosity and nitric oxide binding. **Conclusions:** VBI-1 demonstrates strong potential as a resuscitative fluid, offering blood pressure restoration, reduced oxidative damage, and enhanced tissue perfusion, with significant implications for use in resource-limited and pre-hospital settings.

## 1. Introduction

Exsanguination remains a primary cause of preventable death in both military and civilian contexts, contributing significantly to mortality rates worldwide [1,2,3]. When hemorrhage reaches critical levels, it can quickly lead to clinical death (CD), marked by the absence of a palpable pulse and cessation of respiration.

Hemorrhagic shock from blood loss induces profound systemic effects, including reduced tissue perfusion and impaired oxygen delivery, triggering a cascade of metabolic, immunologic, and hemodynamic changes. Early compensatory responses may briefly sustain perfusion; however, prolonged hemorrhage leads to cellular hypoxia, acidosis, tissue damage, and an exaggerated inflammatory response characterized by pro-inflammatory cytokines and reactive oxygen species (ROS) [4]. This cascade, along with associated coagulopathy, can exacerbate tissue damage, impair organ function, and lead to complications such as multi-organ dysfunction syndrome (MODS) and systemic inflammatory response syndrome (SIRS) and, ultimately, death if untreated [5,6].

Guidelines from the American College of Surgeons Committee on Trauma endorse resuscitation with blood products in all hemorrhagic shock patients to restore circulating blood volume and improve tissue perfusion. Practical limitations arise, particularly in pre-hospital settings where blood is often unavailable. Moreover, solely replacing blood volume does not address the broader pathophysiological impacts of hemorrhagic shock and tissue ischemia, such as metabolic derangements, immune activation, and reperfusion injury once the cause of shock has been addressed. Thus, an ideal resuscitative fluid would not only restore blood volume and circulation but also limit inflammation, avoid worsening coagulopathy, and mitigate end-organ damage [7,8].

While colloids like albumin, hydroxyethyl starch (HES), dextran, and gelatin have been explored for volume resuscitation, each has limitations including cost, risk of adverse reactions, and potential for exacerbating coagulopathy or renal dysfunction [9,10,11,12]. Crystalloids, including saline and lactated Ringer’s, are safer initial options but are often insufficient to achieve long-term hemodynamic stability after severe blood loss. Our previous work with Ringer’s lactate, for instance, showed restored signs of life after intra-arterial infusion of Ringer’s lactate [13] but inadequate blood pressure for sustained survival after severe hemorrhage.

In response to these challenges, we developed novel phospholipid nanoparticle-based resuscitative fluids, VBI-S and VBI-1. These hydrophobic micelles and liposomes offer a promising approach by redistributing nitric oxide concentration, which becomes overproduced in hemorrhagic shock and can contribute to inflammatory damage, reperfusion injury, and persistent hypotension. By controlling NO distribution and release, these PNPs aim to restore survivable blood pressure, improve tissue perfusion and oxygenation, reduce ischemia–reperfusion injury, and support end-organ recovery [14,15].

Given the multifaceted demands of resuscitation in hemorrhagic shock, careful consideration of fluid choice is essential. This study aims to evaluate the effectiveness of these new colloids that expand volume and redistribute nitric oxide in a model of severe hemorrhagic shock.

## 2. Materials and Methods

### 2.1. Ethical Approval

The animal experiments followed the “Guide for the Care and Use of Laboratory Animals” published by the National Academy Press [16]. Ethics approval was obtained from the Institutional Animal Care and Use Committee (IACUC) at the University of Missouri, Kansas City, under Protocol Number 2108.

### 2.2. Development of the Phospholipids Nanoparticle (VBI-1 and VBI-S)

**Figure 1:**
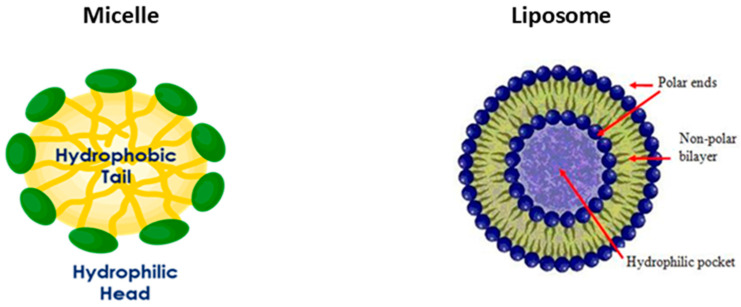
Micelle and Liposome.

We have developed VBI-1 and VBI-S colloids, which are volume expanders that restore blood volume but are also designed to redistribute nitric oxide. Both are composed of micelles and phospholipids. The micelle is composed of a droplet of soybean oil (yellow) (Figure 1) stabilized by phospholipids. The lipophilic segments of the phospholipids (orange) are embedded in the hydrophobic soybean oil. The hydrophilic segment (green) (Figure 1) is in the water that surrounds the micelle. The liposome is composed of a lipid bilayer formed by the phospholipids. When these colloids are infused a hydrophobic space is created. NO is a lipophilic molecule and preferentially localizes into the hydrophobic oil of the micelle and the hydrophobic lipid bilayer of the liposomes. This process reduces the concentration of NO where it is overproduced without interfering with its production or any of its essential autocrine or paracrine interactions [17,18]. 

Using electron microscopy, it was found that the mean particle diameter of VBI-S is 51 nm, and that of VBI-1 is 17 nm. Both VBI-S and VBI-1 absorb NO. However, VBI-S releases NO, while VBI-1 does not.

### 2.3. Experimental Protocol

#### 2.3.1. Hemorrhagic Shock-Induced Clinical Death and Reanimation

To study the most severe form of hemorrhagic shock, we developed the Femoral Artery Catheterization and Reanimation Technique (FACART) to induce clinical death. In our experiment, mean arterial blood pressure dropped below 15 mmHg, characterized by cessation of breathing and palpable pulses. Reanimation was achieved through femoral intra-arterial (IA) fluid infusion.

This study involved two phases with Sprague Dawley rats (n = 6 per group equally distributed by gender). Phase one, lasting 4 h, included four groups: blood, Ringer’s lactate, VBI-S, and VBI-1. Phase two, lasting 12 h, comprised sham, blood, and VBI-1 groups. The sham group underwent general anesthesia for 12 h with femoral artery cannulation but without blood removal or fluid infusion. In the other four groups, blood was removed to induce clinical death. This was approximately 40–45% of all rats’ estimated blood volume. Within one minute after cessation of respiration, the shed blood (experimental group 1) or an equal volume of either Ringer’s lactate (experimental group 2), VBI-S (experimental group 3), or VBI-1 (experimental group 4) was IA-infused.

After pre-procedural weight measurement, rats were anesthetized with 4% isoflurane and maintained on a heating pad to ensure normothermia. Bilateral femoral vessels were surgically accessed, and a catheter was inserted into one femoral artery for blood pressure monitoring. Blood was withdrawn from the contralateral artery until spontaneous respiration ceased and the amount of withdrawn blood was recorded. Then depending on which experimental group, one of the four solutions mentioned above (animal’s own shed blood, LR, VBI-1, or VBI-1) was replaced to reanimate the animal.

#### 2.3.2. Tissue Staining

Tissue processing involves the fixation of heart, lungs, kidneys, liver, and pancreas specimens, followed by embedding and sectioning. Hematoxylin and Eosin staining were used to assess tissue morphology. Immunofluorescent-labeled anti-8-Hydroxy-2′-deoxyguanosine (8-OHdG) antibody was used to detect the effects of free radical damage in the form of DNA damage. Microscopy with a Nikon Eclipse Ti microscope and image processing with ImageJ facilitated data acquisition.

##### Histological Data Quantification and Analyses

The histological analysis included scoring tissue injury severity based on predefined criteria [19,20,21]. Brain immunofluorescence quantification measured total cell fluorescence and Corrected Total Cell Fluorescence (CTCF). Statistical analysis was performed using GraphPad Prism 10, with data presented as mean ± SEM.

#### 2.3.3. Rheology Measurements

Rheology measurements were performed using a Cone and Plate geometry with a 51 µm gap and 2° angle on a stress-controlled Discovery-HR3 rheometer (TA Instruments, Tokyo, Japan). Samples were shaken, loaded, and tested in flow sweep mode to measure viscosity at various shear rates. All measurements were conducted at body temperature (37 °C). To minimize drying, a sealed solvent trap with water was used to maintain the atmosphere around the samples. The flow sweep test was conducted for each sample using steady-state sensing mode for more reliability where each data point was measured over 60 s (recorded every 10 s, totaling six data points). The measurement continued until 3 consecutive data points fell within a 5% tolerance.

## 3. Results

### 3.1. The Femoral Artery Catheterization and Reanimation Techniques Model Simulated Clinical Death and Subsequent Reanimation

(i) Model Development

**Figure 2:**
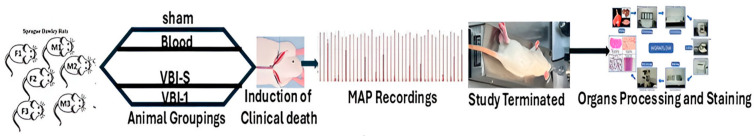
Schematic of the experimental protocol used, before and after the interventions.

**Figure 3:**
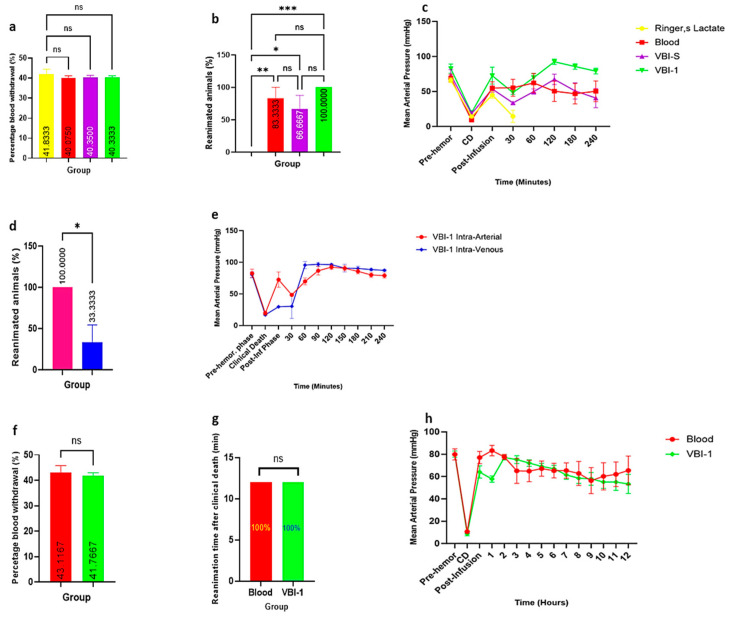
Experiments showing (**a**) the percentage of total blood volume withdrawn, (**b**) the percentage of rats that survived for four hours after reanimation, (**c**) the mean arterial pressure across four treatment groups over 240 min (4 h), (**d**) the percentage of rats that reanimated after intra-arterial versus intra-venous VBI-1 infusion, (**e**) shows the mean arterial pressure of rats that were infused VBI-1 intra-arterially and VBI-1 intravenously over 240 min (4 h), (**f**) the percentage of blood withdrawal, (**g**) the percentage of reanimated animals, and (**h**) the mean arterial pressure across the VBI-1 and blood treatment groups over 12 h. Data were analyzed using one-way ANOVA and Tukey’s post hoc test. * *p* < 0.05, ** *p* < 0.01, *** *p* < 0.001, ns (Not Significant).

Aiming at replicating scenarios of extreme hemorrhage shock, such as those encountered in warfare or other penetrating trauma, we developed a model of clinical death, induced by withdrawing blood until the animal has lost pulses and has no spontaneous respiration. This is usually about 40–45% of the estimated blood volume. Then, depending on which experimental group, the shed blood was replaced by an equal volume of Ringer’s lactate, shed blood, VBI-S, or VBI-1 within 3 min into the femoral artery with subsequent animal reanimation, as shown in Figure 2. There was no significant difference between groups in the amount of blood removed required to induce clinical death (Figure 3a). However, as shown in Figure 3b, LR was notably distinct in the percentage of reanimation that lasted the full four hours (240 min) compared to VBI-1, VBI-S, and shed blood, (VBI-1 *p* = 0.0002, VBI-S *p*= 0.0109, and blood *p* = 0.0015). IA infusion led to the restoration of spontaneous breathing in all groups, but the average survival time for the Ringer’s lactate group was less than 30 min. Of the rats that received shed blood, 83.3% (five out of six) were successfully reanimated and survived for the full 4 h. Similarly, 100% (six out of six) of the rats receiving VBI-1 were reanimated and survived for the full 4-h period; for those infused with VBI-S, 66.6% (four out of six) were reanimated and survived for 4 h. The experiment was concluded at the 4-h mark with euthanasia.

Figure 3c illustrates the changes in mean arterial pressure over the 4 h for rats reanimated after IA infusion. Tukey multiple comparison tests revealed significant differences among groups at various time points following reanimation. At 30 min post-reanimation, the blood group exhibited a statistically significant increase in MAP compared to Ringer’s lactate, VBI-S, and VBI-1. VBI-1 demonstrated a significantly higher MAP (*p* < 0.05) compared to both VBI-S and RL after the 30-min time point. VBI-S showed a significantly higher MAP (*p* < 0.05) compared to RL. Further analysis revealed that at 120 and 150 min post-reanimation, VBI-1 exhibited a significantly higher MAP (*p* < 0.05) compared to both the blood group and VBI-S. Furthermore, a significant elevation in MAP was observed at 180 min post-reanimation in VBI-1 compared to the blood group.

All rats infused with VBI-1 intraarterially were successfully reanimated, achieving a 100% success rate (six out of six), whereas only 33.3% (two out of six) of the IV-infused rats were reanimated for any length of time (Figure 3d). During the initial 30 min post-infusion, the rats receiving IV infusion exhibited a slow elevation in mean arterial pressure. However, this initial lag observed in the IV-infused rats was rectified after 1 h, and subsequently, the MAP values for both routes did not demonstrate any significant difference for the remaining 4-h period of observation (Figure 3e). These data answer the question of whether there is a difference between intra-arterial and intravenous injection of the PNP. 

Due to the satisfactory mean arterial pressure levels observed after the initial 4-h period and the nearly equivalent responses of VBI-1 and shed blood following a single bolus, the decision was made to extend the experiment from 4 to 12 h, focusing solely on comparing VBI-1 and shed blood. The same procedure was performed regarding the removal of blood until clinical death and reanimation with either the shed blood or an equal volume of VBI-1. The animal was then monitored for 12 h instead of 4 and the same tests were performed. 

Figure 3f illustrates that again there was no disparity in the percentage of total blood volume withdrawn to induce CD between the two groups. In Figure 3g, it is observed that all rats (six out of six, 100%) receiving shed blood via the intra-arterial route were successfully reanimated, while five out of six rats given VBI-1 IA were reanimated.

The Tukey multiple comparison tests highlighted an increase in mean arterial pressure (MAP) among rats in both the shed-blood and VBI-1 groups immediately post-infusion (*p* < 0.001) and at 1 h following reanimation (*p* < 0.01) (Figure 3h). Furthermore, a statistically significant elevation in MAP was noted at 3 h post-reanimation in the VBI-1 group compared to the blood group (*p* < 0.05). There was no significant difference between these curves at any other point after the 5-h point (Figure 3h).

### 3.2. The Increased Viscosity of VBI-1 Is Associated with the Sustained Elevated MAP

**Figure 4:**
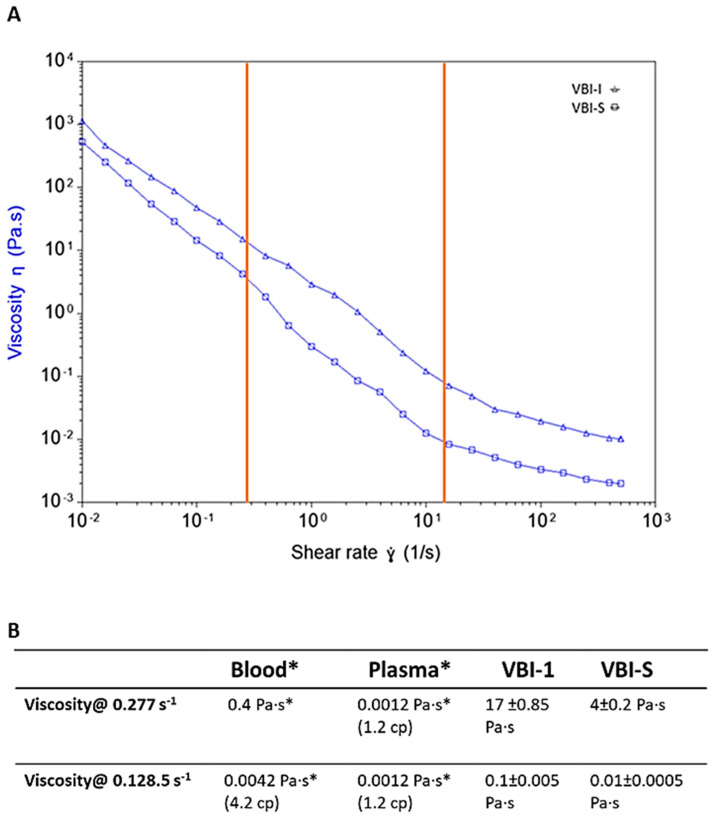
(**A**) Flow Sweep Measurement to measure the viscosity at varying shear rates (deformation speed). Solid orange lines indicate 0.277 s^−1^ and 128.5 s^−1^ shear rates; (**B**) measured viscosity of VBI-1 and VBI-S compared to blood and plasma viscosity. * Values for plasma and blood are taken from [24,25].

Tissue perfusion is a critical determinant of tissue oxygenation which is affected by different parameters such as intravascular volume, blood pressure, and cardiac output. Additionally, Cabrales et al. [22,23] demonstrated that blood pressure increased with blood viscosity. So, in this study, a rheology test with flow sweep mode was used to measure the viscosity of the samples. The blood viscosity data are typically presented at the lowest (0.277 s^−1^) and highest (128.5 s^−1^) shear rates studied since there is a fall in blood viscosity at shear rates above 5 s^−1^ due to red cell deformation and at lower shear rates due to red cell aggregation [24]. Measured shear rates are shown with orange lines in Figure 4A. At a shear rate of 0.277 s^−1^, viscosity was found to be 17 Pa·s for VBI-1 and 4 Pa·s for VBI-S. In comparison, the viscosity of rat blood was reported as 0.4 Pa.s and that of plasma 0.0012 Pa·s (Figure 4B). At the higher shear rate (128.5 s^−1^), viscosity measured 0.1 Pa.s for VBI-1 and 0.01 for VBI-S, while it was 0.0042 Pa·s and 0.0012 Pa·s for blood and plasma, respectively [25]. Therefore, both VBI-1 and VBI-S samples exhibit shear-thinning behavior, akin to blood, where their viscosity decreases as the shear rate (or deformation speed) increases. In contrast, the viscosity of plasma is independent of the shear rate. The higher viscosity of VBI-1 corresponds with the increased blood pressure observed after reanimation, compared to blood. The decrease in viscosity with an increase in shear rate would facilitate the flow of VBI-1 through the microcirculation. The low viscosity of plasma is consistent with the inability to achieve acceptable blood pressure by infusing plasma after severe blood loss [26].

### 3.3. Protective Effects of VBI-1 Against Ischemia–Reperfusion Injury

(i) Histopathological evaluation of Ischemia–Reperfusion Injury

**Figure 5:**
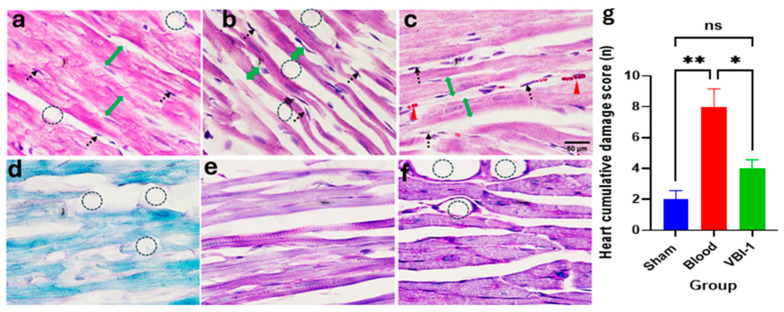
Representative confocal images depict H&E (**a**–**c**) and PAS (**d**–**f**) staining the heart. (×100 (**a**–**c**), ×400 (**d**–**f**)). Sham samples are shown in panels (**a**,**d**), blood samples in panels (**b**,**e**), and VBI-1 samples in panels (**c**,**f**). The histopathological evaluation included an assessment of nuclear pyknosis (black dotted arrow), hemorrhage/congestion (red arrowhead), edema (dotted circle), and hypotrophy (double green arrow in panel (**b**). Sample size (n) is six. Scale bars represent 50 μm. Histopathology quantification (**g**) was conducted using a one-way analysis of variance (ANOVA) with post hoc Dunnett adjustment. The data are presented as mean  ±  SEM. * *p* < 0.05, ** *p* < 0.01, *p* > 0.05 (ns).

Confocal microscopy images of Hematoxylin and Eosin (H&E) staining (Figure 5a–c) and Periodic Acid–Schiff (PAS) staining (Figure 5d–f) were acquired for the heart, lung, liver, kidney, and pancreas tissues after the 12-h experimental period. The pathological scores were evaluated using the ordinal types of data measurements as shown in Table 1.

**Figure 6:**
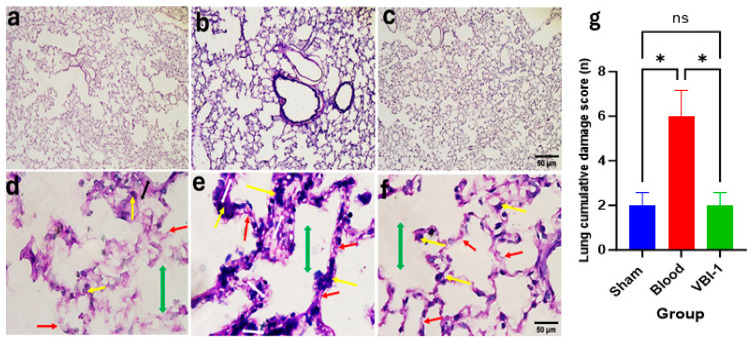
Representative confocal images depict H&E (**a**–**c**) and PAS (**d**–**f**) staining the lung. Magnification (×100 (**a**–**c**), ×400 (**d**–**f**)). Sham samples are shown in panels (**a**,**d**), blood samples in panels (**b**,**e**), and VBI-1 samples in panels (**c**,**f**). The histopathological evaluation included an assessment of tissue integrity (see panel (**b**)), nuclear damage (yellow arrow), fibrosis, and inflammation (white double arrow), alveolar space (double green arrow). Sample size (n) is 6. Scale bars represent 50 μm. Histopathology quantification (**g**) was conducted using a one-way analysis of variance (ANOVA) with post hoc Dunnett adjustment. The data are presented as mean ±  SEM. * *p* < 0.05, *p* > 0.05 (ns).

**Figure 7:**
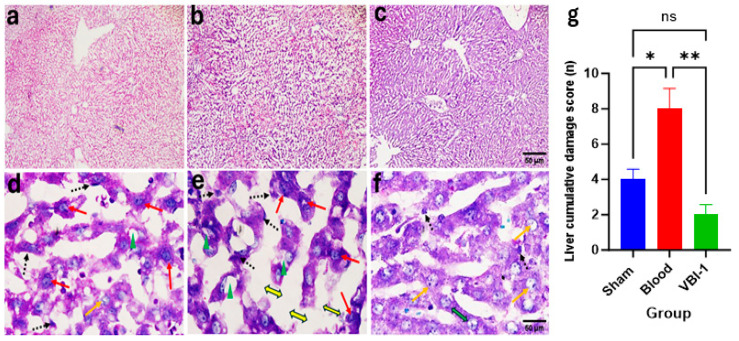
Representative confocal images depict H&E (**a**–**c**) and PAS (**d**–**f**) staining in the liver. Magnification (×100 (**a**–**c**), ×400 (**d**–**f**)) sham samples are shown in panels (**a**,**d**), blood samples in panels (**b**,**e**), and VBI-1 samples in panels (**c**,**f**). The histopathological evaluation included an assessment of nuclear pyknosis (black dotted arrow), nuclear damage (red arrow), hepatocyte vacuolization (green arrowhead), and tissue degeneration (yellow double arrow). Sample size (n) is 6. Scale bars represent 50 μm. Histopathology quantification (**g**) was conducted using a one-way analysis of variance (ANOVA) with post hoc Dunnett adjustment. The data are presented as mean  ±  SEM. * *p* < 0.05, ** *p* < 0.01, *p* > 0.05 (ns).

**Figure 8:**
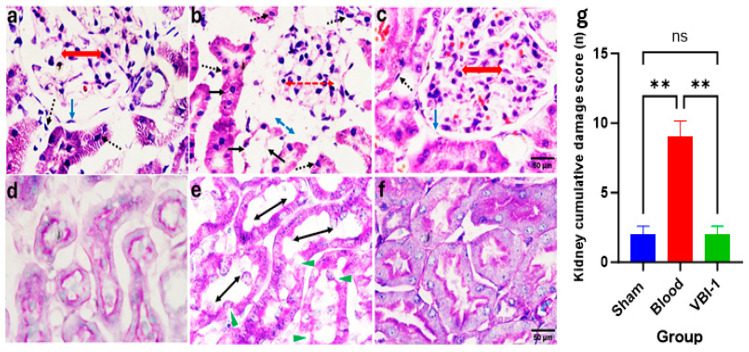
Representative confocal images depict H&E (**a**–**c**) and PAS (**d**–**f**) staining the kidney. Magnification (×100 (**a**–**c**), ×400 (**d**–**f**)). Sham samples are shown in panels (**a**,**d**), blood samples in panels (**b**,**e**), and VBI-1 samples in panels (**c**,**f**). The histopathological evaluation included an assessment of nuclear pyknosis (blue arrow), nuclear damage (blue broken arrow), tubule vacuolization (green arrowhead), hemorrhage (see panel (**c**)), corpuscular damage (blue and red double arrows), and widened tubule lumen (black double arrow). Sample size (n) is 6. Scale bars represent 50 μm. Histopathology quantification (**g**) was conducted using a one-way analysis of variance (ANOVA) with post hoc Dunnett adjustment. The data are presented as mean  ±  SEM. ** *p* < 0.01, *p* > 0.05 (ns).

**Figure 9:**
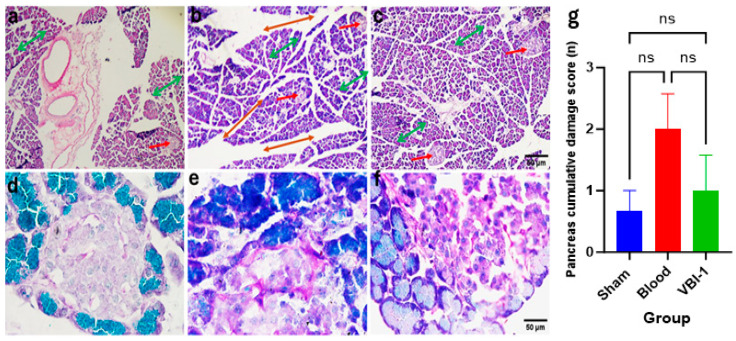
Representative confocal images depict H&E (**a**–**c**) and PAS (**d**–**f**) staining in the pancreas. Magnification ((×100 (**a**–**c**), ×400 (**d**–**f**)). Sham samples are shown in panels (**a**,**d**), blood samples in panels (**b**,**e**), and VBI-1 samples in panels (**c**,**f**). The histopathological evaluation included an assessment of Acini (green double arrow), Islet of Langerhans (red arrow), nuclear damage, and septa thickening (red double arrow). Sample size (n) is 6. Scale bars represent 50 μm. Histopathology quantification (**g**) was conducted using a one-way analysis of variance (ANOVA) with post hoc Dunnett adjustment. The data are presented as mean ± SEM. *p* > 0.05 (ns).

Our findings revealed a substantial increase in the cumulative damage score, indicative of pathological lesions, across all examined tissues in rats subjected to shed-blood infusion compared to those infused with VBI-1 or subjected to the sham procedure in which rats were given anesthesia and subjected to bilateral femoral artery cannulation without withdrawal or injection of fluid. Notable histological lesions observed included nuclear damage, myocyte vacuolization, widened inter-myofiber spaces, and edema in the cardiac tissue (Figure 5, Table 2), as well as nuclear damage, pneumocyte vacuolization and fibrosis in the lung tissue (Figure 6, Table 3). Furthermore, nuclear damage, tissue vacuolization, hepatocyte vacuolization, and tissue degeneration were evident in the hepatic tissue (Figure 7, Table 4), while nuclear damage, tubule vacuolization, hemorrhage, corpuscular damage, and tubule damage were observed in the renal tissue (Figure 8, Table 5). Additionally, nuclear damage and acini shrinkage were noted in the pancreatic tissue (Figure 9, Table 6).

(ii) VBI-1 mitigates oxidative stress-induced damage and DNA injury.

**Figure 10:**
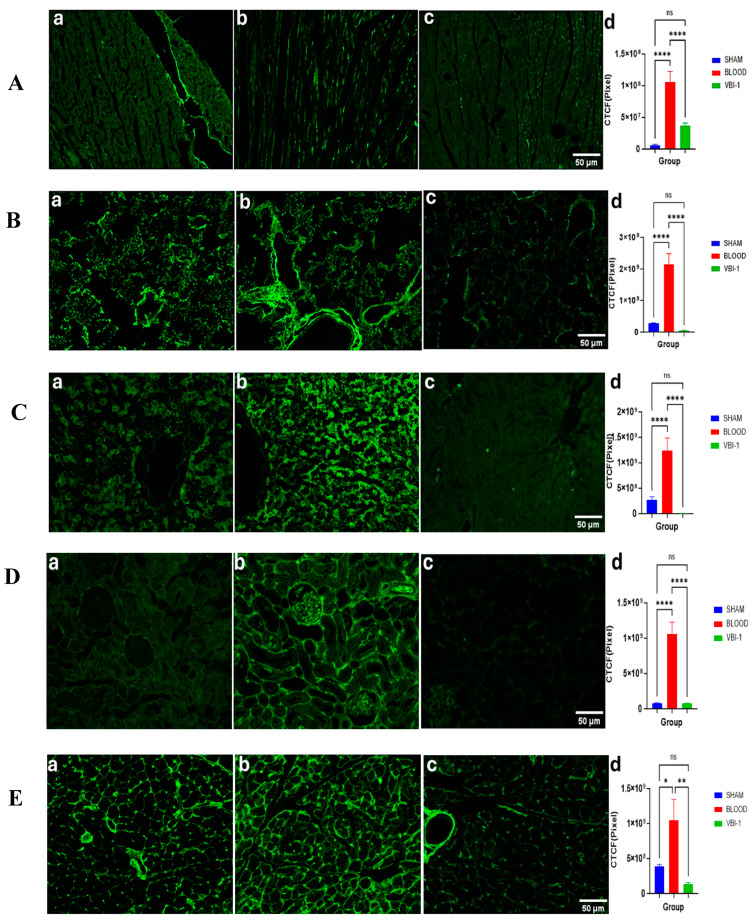
Representative confocal images of immunofluorescence staining for oxidative stress and DNA damage (8-Hydroxyguanosine), magnification X100. in the heart (**A**), lungs (**B**), liver (**C**), kidney (**D**), and pancreas (**E**). Sham (**a**), blood (**b**), VBI-1 (**c**). n = 4. Quantified fluorescence was analyzed using a one-way analysis of variance (ANOVA) with post hoc Dunnett adjustment (**d**). The data are presented as mean ± SEM. *p* > 0.05 (ns), * *p* < 0.05, ** *p* < 0.01, **** *p* < 0.0001.

To investigate the protective role of VBI-1 against reperfusion injury, we assessed the expression of 8-hydroxyguanosine using immunofluorescence staining. This analysis aimed to elucidate the mechanisms underlying oxidative stress-induced damage and DNA injury. Our results demonstrated an upregulation of 8-OHdG expression in all examined tissues of rats subjected to shed-blood infusion, in contrast to those infused with VBI-1 or subjected to the sham procedure (Figure 10).

### 3.4. VBI-1 Modulation of Nitric Oxide

**Figure 11:**
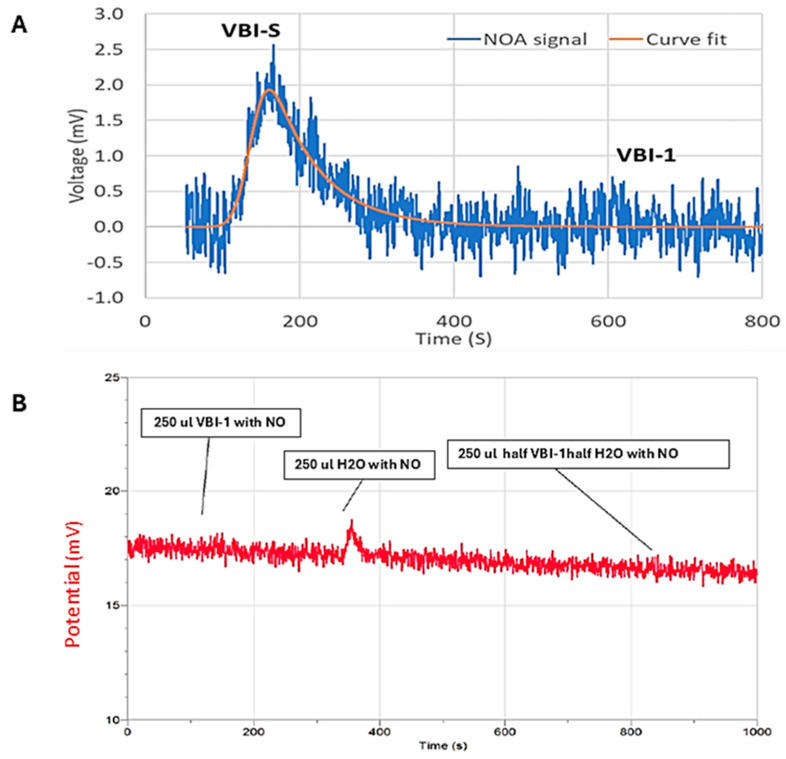
(**A**) Representative example of the interaction of VBI-S and VBI-1 with NO measured by chemiluminescence; (**B**) the peak on the left is that of NO uptake by water. To the right one sees that there is no uptake of NO by a mixture of water and VBI-1, suggesting that the binding of NO with VBI-1 is irreversible.

Knowing the microcirculatory effects of NO in states of ischemia and shock, we proceeded to investigate the interaction between VBI-1 and NO as well as VBI-S and NO. The outcomes of this experiment are depicted in Figure 11A. A prominent peak on the left indicates the interaction of NO with VBI-S. In this experiment, VBI-S was isolated in a NO-enriched environment for two minutes. Subsequently, 500 µL of the NO-saturated VBI-S was introduced into the sample chamber, followed by the displacement of NO from VBI-S with a continuous stream of pure helium gas. The graph illustrates an increase in NO leaving VBI-S, succeeded by a decline. A similar procedure was conducted with VBI-1; however, no peak was detected. The absence of a peak in VBI-1 could be attributed to either the lack of interaction with NO or the uptake of NO by VBI-1 without subsequent release, contrary to VBI-S, where both uptake and release occurred. To help differentiate between these possibilities, the NO content of water was measured using the same methodology. If VBI-1 were not interacting with NO, one would anticipate observing a peak resembling that of water upon dilution. Figure 11B shows the peak observed with pure water. In a separate sample, 1 mL of VBI-1 was diluted with 6 mL of water, so that its water content now approached 95% (i.e., essentially identical to the pure water sample). However, Figure 11B illustrates that even after dilution, no peak derived from water was observed with VBI-1. This outcome aligns with the hypothesis of an irreversible uptake of NO by VBI-1.

## 4. Discussion

The clinical scenario modeled by our experiment is that of the hemorrhagic shock of a civilian or warfighter ranging from significant blood loss to clinical death—pulseless and apneic. In our rat model of CD, we demonstrated the efficacy of the infusion of VBI-1 via the femoral artery in restoring spontaneous respiration and elevating blood pressure. 

While venous infusion is typically preferred for fluid administration, our findings show that for restoration of breathing and sustained survivable blood pressure, IA infusion is superior to IV infusion in states of CD. Nonetheless, our findings have shown that 33% of clinically dead animals receiving IV infusion will reanimate, making this a viable alternative if the artery cannot be accessed.

To elucidate the mechanisms underlying VBI-1’s efficacy further, we examined its impact on viscosity and nitric oxide modulation. Even though the particles of VBI-1 are much smaller than red blood cells, VBI-1 elevated blood pressure as well as blood. The fact that VBI-1 is much more viscous than blood could be the reason for this equivalence. In fact, our results demonstrated VBI-1’s superiority over blood in elevating blood pressure within the initial four hours post-reanimation, with sustained effectiveness over 12 h. This elevation is attributed to VBI-1’s multifaceted mechanism, which involves increased viscosity and modulation of NO dynamics and volume expansion. We do not think that VBI-1 is causing vasoconstriction. We did not see any evidence of ischemia in the heart or any other organ although 40–45% of the blood volume was replaced with VBI-1. Had VBI-1 acted as a vasoconstrictor, we would expect to observe ischemic lesions due to diminished blood flow. We think that ischemia did not occur because VBI-1 is not a high-affinity NO scavenger like hemoglobin [27]. Our observations are consistent with the possibility that even at high blood replacement with VBI-1, VBI-1 only reduces excessive levels of NO and does not interfere with basal or homeostatic levels of NO. This uptake of NO could have contributed to the increase in MAP after infusion of VBI-1 by normalizing vascular diameter and reducing cardiac exposure to NO. Also consistent with the elevation of MAP by VBI-1 was its viscosity. We found the viscosity of VBI-1 to be significantly higher than that of rat blood and plasma, emphasizing its crucial role in maintaining tissue perfusion and organ function, particularly in hemorrhagic shock. This finding highlights the dynamic impact of VBI-1 on blood viscosity and microcirculatory blood flow, which are crucial factors in maintaining tissue perfusion and organ function. The elevated viscosity of VBI-1 refers to its ability to increase the thickness or resistance to the flow of blood. This elevated viscosity is beneficial in the context of hemorrhagic shock because it promotes the generation of initial high blood pressure before the blood enters the microcirculation. When blood leaves the heart and enters larger arteries, the increased viscosity of VBI-1 aids in generating higher pressure, ensuring adequate perfusion to vital organs despite the reduced blood volume due to hemorrhage. This initial high pressure is essential for maintaining organ perfusion and preventing tissue hypoxia. As blood flows from larger arteries into smaller arterioles and capillaries within the microcirculation, the viscosity of VBI-1 decreases. This decrease in viscosity would allow blood or VBI-1 to flow more freely through the narrow capillaries, enhancing microcirculatory flow. Improved microcirculatory flow is critical for delivering oxygen and nutrients to tissues at the cellular level, supporting cellular metabolism, and removing metabolic waste products. 

Furthermore, our assessment of VBI-1’s protective role in reperfusion injury revealed significant organ protection compared to infusion of blood. This protective effect is attributed to VBI-1’s ability to absorb excessive nitric oxide and free radicals during ischemia–reperfusion injury. Our data show that VBI-1 demonstrates remarkable efficacy in absorbing excessive nitric oxide. There is evidence also that phosphatidylcholine, a component of VBI-1, is a scavenger of superoxide [28].

Nitric oxide reacting with superoxide to produce peroxynitrite and other free radicals is known to exacerbate tissue damage and oxidative stress during ischemia–reperfusion events [29]. By absorbing these harmful molecules, VBI-1 protects against tissue injury, reducing organ failure. Our analysis of oxidative stress-induced DNA damage indicated a significant reduction of 8-hydroxyguanosine following VBI-1 infusion, highlighting its potent antioxidant properties and protective effects on cellular DNA. This finding demonstrated that infusion of VBI-1 has a protective effect on reperfusion injury to the organs studied. 8-hydroxyguanosine is formed upon DNA repair that occurs due to oxidative nuclear and mitochondrial DNA damage due to free radical attack on cellular macromolecules. Elevated levels of 8-OHdG indicate increased oxidative stress within cells, which can lead to cellular dysfunction or death. By scavenging reactive oxygen species or modulating oxidative stress pathways, VBI-1 can protect cellular DNA from oxidative damage after restoration of blood flow.

There are many end-organ effects associated with blood transfusion including potentially life-threatening pulmonary decompensation [30,31,32,33,34]. The mortality rate when blood transfusion triggers acute respiratory distress syndrome is 35–42% [35]. The histological sections shown in this article show severe lung injury after only one transfusion of the rat’s own blood. If VBI-1 were used instead of blood or combined with blood, the protection against ischemia and reperfusion injury could reduce the occurrence of this complication. There may be other unknown mechanisms leading to decreased tissue injury as well. Similar strategies could reduce the incidence of injury to other vital organs.

Finally, given the fact that VBI-1 is as effective as blood in elevating blood pressure acutely and long-term, we think it would be possible to routinely use VBI-1 to restore intravascular volume instead of blood for the first 40% of the blood volume. The protective effect of VBI-1 in preventing ischemia–reperfusion injury would make it preferable to blood. There are other advantages of VBI-1 over blood such as not needing a type and cross, no screening for pathogens, and its stability at room temperature for at least one year compared to blood’s refrigerated shelf life of only 42 days. If VBI-1 could be used for at least 40% of the initial blood volume, then problems with the world’s blood supply would be greatly alleviated including for warfighters, in the pre-hospital setting, or with difficulty of access to blood products. We will explore this and other possibilities in our future research.

## 5. Conclusions

In conclusion, the multifaceted mechanisms of action displayed by VBI-1, encompassing its functions as a volume expander, NO and free radical scavenger, oxygen carrier, and enhancer of microcirculatory flow underscore its promising role as a therapeutic agent alleviating tissue damage and facilitating recovery in ischemia–reperfusion injuries, whenever tissue perfusion is restored. Further investigation into the specific pathways through which these effects occur, as well as their clinical implications, is imperative to fully exploit the therapeutic potential of VBI-1 in ischemic conditions and after severe blood loss.

## Figures and Tables

**Table 1:** Ordinal types of histological scoring criteria.

Score	Description
0	Normal
1	Minimal
2	Moderate
3	Severe

**Table 2:** Heart histopathologic scores.

Features	Sham	Blood	VBI-1
Nuclear Damage	1	2	1
Myofiber atrophy	0	3	1
myocardial interstitial edema.	1	3	2
Cumulative score	2	8	4

**Table 3:** Lung histopathologic scores.

Features	Sham	Blood	VBI-1
Nuclear Damage	1	3	1
Septal Thickening	1	2	1
Cumulative Score	2	5	2

**Table 4:** Liver histopathologic scores.

Features	Sham	Blood	VBI-1
Nuclear Damage	2	2	1
Tissue Vacuolation	1	2	1
Hepatocytes Vacuolation	1	2	0
Cumulative score	4	6	2

**Table 5:** Kidney histopathologic scores.

Features	Sham	Blood	VBI-1
Nuclear Damage	1	2	1
Tubular damage	1	2	0
Tubular Vacuolation	0	3	0
Glomerular degeneration	0	2	0
Hemorrhage	0	0	1
Cumulative score	2	9	2

**Table 6:** Pancreas histopathologic scores.

Features	Sham	Blood	VBI-1
Islet cells derangements	0	1	1
Acini shrinkage	0	1	0
Cumulative score	0	2	1

## Data Availability

All data were made available in the publication.

## References

[1] Eastridge B.J., Holcomb J.B., Shackelford S. (2019). Outcomes of traumatic hemorrhagic shock and the epidemiology of preventable death from injury. Transfusion.

[2] Kalkwarf K.J., Drake S.A., Yang Y., Thetford C., Myers L., Brock M., Wolf D.A., Persse D., Wade C.E., Holcomb J.B. (2020). Bleeding to death in a big city: An analysis of all trauma deaths from hemorrhage in a metropolitan area during 1 year. J. Trauma Acute Care Surg..

[3] Duchesne J., Taghavi S., Houghton A., Khan M., Perreira B., Cotton B., Tatum D., Damage Control Resuscitation Committee (2021). Prehospital Mortality Due to Hemorrhagic Shock Remains High and Unchanged: A Summary of Current Civilian EMS Practices and New Military Changes. Shock.

[4] Kuo K., Palmer L. (2022). Pathophysiology of hemorrhagic shock. J. Vet. Emerg. Crit. Care.

[5] Dufour-Gaume F., Frescaline N., Cardona V., Prat N.J. (2023). Danger signals in traumatic hemorrhagic shock and new lines for clinical applications. Front. Physiol..

[6] Gutierrez G., Reines H.D., Wulf-Gutierrez M.E. (2004). Clinical review: Hemorrhagic shock. Crit. Care.

[7] Chipman A.M., Jenne C., Wu F., Kozar R.A. (2020). Contemporary resuscitation of hemorrhagic shock: What will the future hold?. Am. J. Surg..

[8] Aksu U., Bezemer R., Yavuz B., Kandil A., Demirci C., Ince C. (2012). Balanced vs. unbalanced crystalloid resuscitation in a near-fatal model of hemorrhagic shock and the effects on renal oxygenation, oxidative stress, and inflammation. Resuscitation.

[9] Chang R., Holcomb J.B. (2017). Optimal Fluid Therapy for Traumatic Hemorrhagic Shock. Crit. Care Clin..

[10] White N.J., Ward K.R., Pati S., Strandenes G., Cap A.P. (2017). Hemorrhagic blood failure: Oxygen debt, coagulopathy, and endothelial damage. J. Trauma Acute Care Surg..

[11] Hilbert-Carius P., Schwarzkopf D., Reinhart K., Hartog C.S., Lefering R., Bernhard M., Struck M.F. (2018). Synthetic colloid resuscitation in severely injured patients: Analysis of a nationwide trauma registry (TraumaRegister DGU). Sci. Rep..

[12] Kheirabadi B.S., Valdez-Delgado K.K., Terrazas I.B., Miranda N., Dubick M.A. (2015). Is limited prehospital resuscitation with plasma more beneficial than using a synthetic colloid? An experimental study in rabbits with parenchymal bleeding. J. Trauma Acute Care Surg..

[13] Youssef A.M., Hamidian Jahromi A., Simpkins C.O. (2016). Arterial versus venous fluid resuscitation; restoring cardiac contractions in cardiac arrest following exsanguinations. Trauma Mon..

[14] Zhao Y., Ouyang X., Peng Y., Peng S. (2021). Stimuli-Responsive Nitric Oxide-Based Nanomedicine for Synergistic Therapy. Pharmaceutics.

[15] Andrabi S.M., Sharma N.S., Karan A., Shahriar S.M.S., Cordon B., Ma B., Xie J. (2023). Nitric Oxide: Physiological Functions, Delivery, and Biomedical Applications. Adv. Sci..

[16] Institute for Laboratory Animal Research (U.S.), Committee on Update of the Guide for the Care and Use of Laboratory Animals., National Research Council (U.S.), Institute for Laboratory Animal Research, National Academies Press (U.S.), National Research Council (U.S.), Institute for Laboratory Animal Research (1996). Guide for the Care and Use of Laboratory Animals.

[17] Shah N.S., Billiar T.R. (1998). Role of nitric oxide in inflammation and tissue injury during endotoxemia and hemorrhagic shock. Environ. Health Perspect..

[18] Collins J.L., Vodovotz Y., Hierholzer C., Villavicencio R.T., Liu S., Alber S., Gallo D., Stolz D.B., Watkins S.C., Godfrey A. (2003). Characterization of the expression of inducible nitric oxide synthase in rat and human liver during hemorrhagic shock. Shock.

[19] Kroemer G., Galluzzi L., Vandenabeele P., Abrams J., Alnemri E.S., Baehrecke E.H., Blagosklonny M.V., El-Deiry W.S., Golstein P., Green D.R. (2009). Classification of cell death: Recommendations of the Nomenclature Committee on Cell Death 2009. Cell Death Differ..

[20] Smith J.K., Johnson L.M., Brown A.B. (2020). Principles for valid histopathologic scoring in research. J. Histopathol. Res..

[21] Andrijevic D., Vrselja Z., Lysyy T., Zhang S., Skarica M., Spajic A., Dellal D., Thorn S.L., Duckrow R.B., Ma S. (2022). Cellular recovery after prolonged warm ischaemia of the whole body. Nature.

[22] Cabrales P., Martini J., Intaglietta M., Tsai A.G. (2006). Blood viscosity maintains microvascular conditions during normovolemic anemia independent of blood oxygen-carrying capacity. Am. J. Physiol. Heart Circ. Physiol..

[23] Cabrales P., Tsai A.G. (2006). Plasma viscosity regulates systemic and microvascular perfusion during acute extreme anemic conditions. Am. J. Physiol. Heart Circ. Physiol..

[24] Johnn H.I., Phipps C.G., Gascoyne S.C., Hawkey C.M., Rampling M.W. (1992). A comparison of the viscometric properties of the blood from a wide range of mammals. Clin. Hemorheol. Microcirc..

[25] Cabrales P. (2009). Low-dose nitrite enhances perfusion after fluid resuscitation from hemorrhagic shock. Resuscitation.

[26] Nader E., Skinner S., Romana M., Fort R., Lemonne N., Guillot N., Gauthier A., Antoine-Jonville S., Renoux C., Hardy-Dessources M.-D. (2019). Blood rheology: Key parameters, impact on blood flow, role in sickle cell disease and effects of exercise. Front. Physiol..

[27] Han T.H., Hyduke D.R., Vaughn M.W., Fukuto J.M., Liao J.C. (2002). Nitric oxide reaction with red blood cells and hemoglobin under heterogeneous conditions. Proc. Natl. Acad. Sci. USA.

[28] Yoshida K., Mohsenin V. (1991). Unsaturated phosphatidylcholines inhibit superoxide production in human neutrophils. Life Sci..

[29] Beckman J.S., Koppenol W.H. (1996). Nitric oxide, superoxide, and peroxynitrite: The good, the bad, and ugly. Am. J. Physiol..

[30] Zilberberg M.D., Carter C., Lefebvre P., Raut M., Vekeman F., Duh M.S., Shorr A.F. (2007). Red blood cell transfusions and the risk of acute respiratory distress syndrome among the critically ill: A cohort study. Crit. Care.

[31] Nossaman B.D. (2008). Transfusion-Related Acute Lung Injury (TRALI): Report of 2 Cases and a Review of The Literature. Ochsner J..

[32] Voelker M.T., Spieth P. (2019). Blood transfusion-associated lung injury. J. Thorac. Dis..

[33] Aubron C., Hourmant B., Menguy J., Sparrow R.L. (2021). Transfusion-related respiratory complications in intensive care: A diagnosis challenge. Transfus. Clin. Biol..

[34] Yu Y., Lian Z. (2023). Update on transfusion-related acute lung injury: An overview of its pathogenesis and management. Front. Immunol..

[35] Sadana D., Kaur S., Sankaramangalam K., Saini I., Banerjee K., Siuba M., Amaral V., Gadre S., Torbic H., Krishnan S. (2023). Mortality associated with acute respiratory distress syndrome, 2009–2019: A systematic review and meta-analysis. Crit Care Resusc..

